# Heat stroke associated with novel leukaemia inhibitory factor receptor gene variant in a Chinese infant

**DOI:** 10.1515/biol-2025-1195

**Published:** 2025-10-30

**Authors:** Yanling Chen, Yumei Liu, Shaoru He, Juan Gui, Yifei Wang, Manli Zheng

**Affiliations:** Department of Neonatology, Guangdong Provincial People’s Hospital (Guangdong Academy of Medical Sciences), Southern Medical University, Guangdong, 510080, China; Department of Neonatology, Guangdong Provincial People’s Hospital (Guangdong Academy of Medical Sciences), Southern Medical University, No. 106 Zhongshan Er Road, Guangdong, 510080, China

**Keywords:** Stüve–Wiedemann, hyperthermia, thermal dysregulation, dysautonomia, pulmonary hypertension

## Abstract

Stüve–Wiedemann syndrome (SWS) is a rare autosomal recessive genetic disorder characterised by skeletal dysplasia, dysautonomia, and multi-system abnormalities. It is typically caused by variants in the leukaemia inhibitory factor receptor (LIFR) gene. This case report presents a novel and complex heterozygous variant in the LIFR gene in a 2-month-old Chinese infant, which contributes to the limited literature on SWS in the Chinese population and underscores the importance of early identification and intervention. The infant was born at 38 weeks of gestation via caesarean section due to breech presentation. He presented with multiple symptoms, including persistent pulmonary hypertension of the newborn, recurrent hyperthermia, and joint deformities. Whole exome sequencing identified a novel compound heterozygous variant in the LIFR gene. The infant underwent various interventions, including mechanical ventilation, inhaled nitric oxide, and nasogastric feeding. Despite these measures, the infant experienced recurrent hyperthermia episodes leading to multi-organ dysfunction. The infant was eventually stabilised, but follow-up revealed global developmental delay and persistent skeletal abnormalities. Early identification of the LIFR gene variant is crucial for timely intervention and management of multi-system complications. Further research is warranted to explore targeted therapies and improve outcomes for patients with this rare disorder.

## 1 Introduction

Stüve–Wiedemann syndrome (SWS) is a rare autosomal recessive genetic disorder typically caused by a variant in the leukaemia inhibitory factor receptor (LIFR) gene [1,2]. The syndrome is characterised by considerable skeletal dysplasia, autonomic nervous disorders, swallowing and eating difficulties, frequent respiratory infections, and other multi-system clinical manifestations. Respiratory distress and hyperthermia episodes are the leading causes of early neonatal death. Thermal dysregulation is particularly prominent in patients with SWS, substantially impacting their quality of life and daily functions. Early recognition and diagnosis may help optimise clinical management. Previous studies on SWS have primarily focused on populations in the Middle East and Europe [3–6]. In this report, we present the case of a 2-month-old Chinese infant diagnosed with SWS, providing a brief clinical course and discussing his case presentation. This case report contributes to the existing literature on thermal dysregulation in paediatric patients with SWS, aiming to enhance awareness of the syndrome and underscore the importance of early identification, intervention, and genetic counselling for families. Ultimately, these efforts will contribute to an improved understanding of the condition and better health outcomes for affected children.

## 2 Case report

The infant, born at 38 weeks via caesarean section due to breech presentation, experienced respiratory distress and was admitted to the neonatal department. He had persistent hypoxaemia requiring mechanical ventilation and other treatments. He was diagnosed with persistent pulmonary hypertension of the newborn (PPHN) and received high-frequency ventilation, inhaled nitric oxide (iNO), milrinone, and other supportive therapies, which led to gradual clinical stabilisation. Post-extubation, he had feeding difficulties and aspiration pneumonia requiring nasogastric feeding. Despite aEEG showing developmental delay and episodes of fever, no infection was found. The family declined whole-exome sequencing. The infant was discharged at 30 days after the family learned nasogastric feeding and sputum suctioning skills.

On the day of discharge, the infant developed a high fever of 41°C after prolonged swaddling and crying, leading to respiratory distress, poor responsiveness and increased muscle tension. Despite initial interventions at the local hospital, his condition remained critical. Upon readmission to our hospital, he presented with high fever, respiratory distress, hypotension, and liver failure with coagulation disorders. After treatment, including hepatoprotective measures and immunoglobulin support, he gradually improved. He also experienced afebrile seizures managed with anticonvulsants. Central fever was suspected due to recurrent fevers without signs of sepsis or infection.

**Figure 1:**
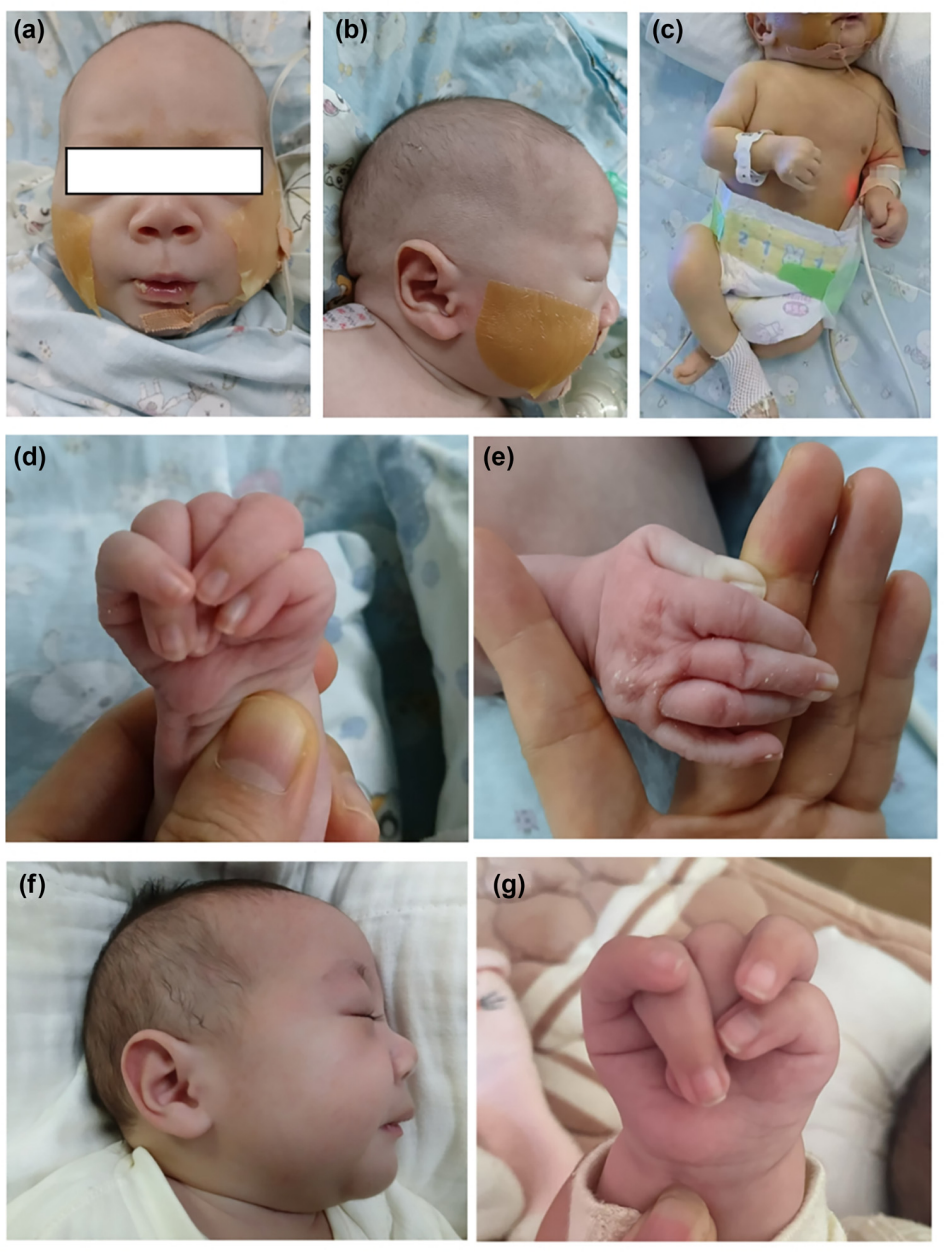
Clinical features and skeletal abnormalities in the infant. (a) and (b) The child has facial abnormalities: A narrow face, swollen eyes, and low-set ears. Due to feeding difficulties, a nasogastric feeding tube has been placed. (c) The lower limbs were short and bent. (d) Abnormal finger posture with flexion contracture of the fingers. (e) The finger joints were flexed and extension was limited. (f) Low-set ears at 6 months of age. (g) Fingers remained in flexion contracture at 6 months of age.

**Figure 2:**
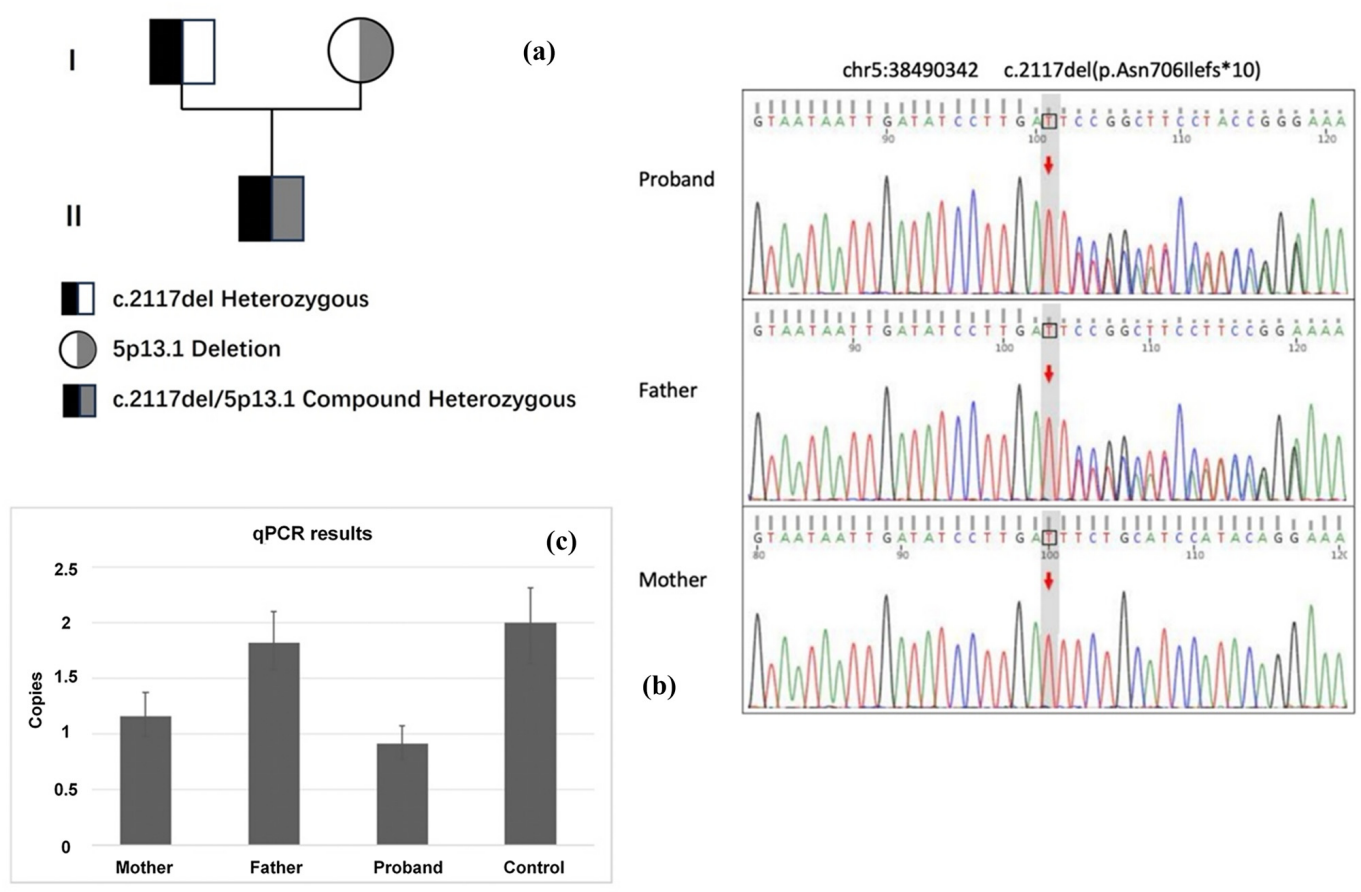
Genetic findings in the proband and family. (a) Pedigree of the family. (b) and (c) Proband carried a heterozygous c.2117del (p.Asn706Ilefs*10) variant inherited from the father, and a heterozygous deletion involving exons 17–18 on the other allele, inherited from the mother. These two variants were confirmed by Sanger sequencing and quantitative PCR, respectively.

The infant presented with dolichocephaly, low-set ears, high palate arch (Figure 1a, b and f), limited joint movement and short, flexed lower limbs (Figure 1c, d and e). Prenatal ultrasound revealed femur and humerus lengths below standard deviations. Whole-exome gene testing identified a compound heterozygous variant in the LIFR gene: a frameshift variant from the father (c.2117del, Figure 2) and a 2 kb heterozygous deletion from the mother involving exons 17–18 and introns. These variants, not previously reported, are likely to cause loss of normal protein function. Considering the infant’s oligohydramnios, PPHN, feeding difficulties, abnormal neurodevelopment, thermoregulation issues, and finger joint deformity, a diagnosis of SWS was established. Impaired thermoregulation was recognised as a dysautonomia manifestation in SWS.

**Figure 3:**
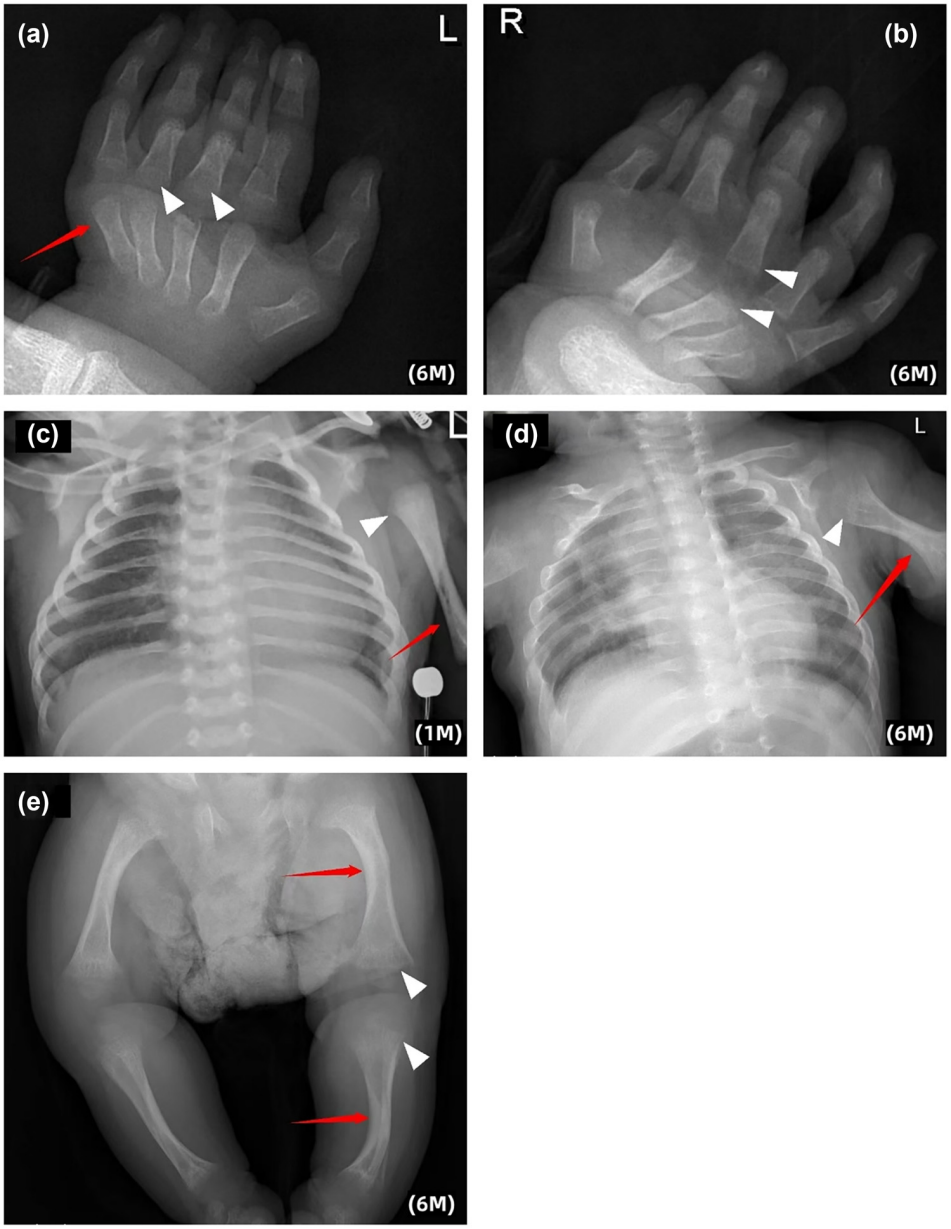
Radiological evidence of skeletal dysplasia. (a) and (b) Trabecular bone of the metacarpal and the epiphysis of fingers on both sides were sparse and blurred (▲). Metacarpophalangeal and interphalangeal joints flex toward the volar side (↑). (c) and (d) Bowing of the long bones (↑) and the enlargement of the humerus metaphysis (▲) was more obvious at 6 months than at 1 month. (e) Bowing of the long bones, widening of femoral and tibial metaphysis with blurred margins (▲), mild cortical thickening and abnormal trabecular pattern in the long bones were also noted (↑).


Following discharge, the patient continued nasogastric feeding due to poor swallowing and sucking abilities and the risk of aspiration. The family followed guidance to prevent hyperthermia by avoiding over-wrapping. The child had one episode of upper respiratory tract infection with high fever but no severe dehydration or organ damage. At 6 months, the child showed global developmental delay with weight and height below −3 SD. Gesell Developmental Scales indicated severe to profound delay (DQ scores 19–35). Persistent flexion contractures of the interphalangeal joints were noted (Figure 1g), and X-ray imaging revealed increased curvature of long bones, enlarged epiphyses, osteoporosis, and mild cortical thickening, indicating abnormal skeletal development (Figure 3a–e).

**Informed consent:** Informed consent has been obtained from legal guardians of the individual included in this study.
**Ethical approval:** The research related to human use has been complied with all the relevant national regulations, institutional policies and in accordance with the tenets of the Helsinki Declaration, and has been approved by the Ethics Committee at Guangdong Provincial People’s Hospital (approval number KY2025-072-01).

## 3 Discussion

SWS is a rare genetic disorder, previously mainly reported in the Middle East and Europe and more common in the United Arab Emirates due to consanguineous marriages. It was first described in 1971 [2]. The condition is characterised by congenital curvature of long bones and fingers, dyspnoea, dysautonomia, and other malformations. It is caused by variants in the LIFR gene on chromosome 5p13.1 [7], often leading to neonatal or early infant death. A 2022 meta-analysis of 69 children with SWS showed a median age of 32 months [3]. Prenatal manifestations included short and curved long bones (32%), intrauterine growth restriction (17%), and oligohydramnios (16%). These non-specific signs should prompt early identification of SWS before birth. The infant in this case developed progressive dyspnoea likely due to severe PPHN, with initial concerns of pulmonary dysplasia due to low amniotic fluid. However, stabilisation was achieved through high-frequency ventilation, iNO, and pressor therapy, allowing respiratory support withdrawal. Patients with SWS have high early mortality, with pulmonary hypertension being a major poor prognostic factor (mortality rate 63%) [1]. Rass-Rothschild et al. reported siblings with SWS with severe pulmonary hypertension and abnormal pulmonary artery development, speculating that early ductus arteriosus closure may increase right ventricular load [8]. This highlights the need for comprehensive cardiovascular assessment in SWS, with further research on cardiovascular mechanisms recommended.

SWS belongs to the family of ciliary neurotrophic factor receptor (CNTFR) pathway-related diseases due to autonomic dysfunction linked to the CNTFR gene [9–12]. This dysfunction arises from impaired cardiomyocyte-like cytokine factor 1 (CLCF-1) signalling caused by LIFR gene variants that disrupt the CLCF-1/CRLF-1 receptor complex [13]. Similar to Crisponi syndrome, which involves variants in CRLF1 or CLCF1 genes [14], SWS shares overlapping clinical features but has distinct genetic origins. Molecular evaluation helps differentiate SWS from other syndromes with similar presentations, aiding in better understanding and management.

The hallmark features of SWS include profound skeletal abnormalities such as bowed long bones, joint contractures, osteoporosis, talipes valgus, flared iliac wings, spinal deformities, hypoplastic lower ilia, micrognathia, and generalised hypotonia. These manifestations tend to progress with age and become more pronounced after age two, often requiring coordinated multidisciplinary intervention including orthopaedic, respiratory, and rehabilitative care [1]. Most SWS cases result from biallelic LIFR gene variants, which are believed to impair sympathetic neuron survival and motor neuron innervation, contributing to both the skeletal phenotype and autonomic instability [7]. Thermal dysregulation in SWS has been linked to impaired JAK/STAT3 pathway signalling, which also affects immune function, thereby increasing susceptibility to infection [15]. However, some patients with SWS lack LIFR variants, indicating genetic heterogeneity. Another gene, GP130 (IL6ST), was recently identified in a series of patients with SWS (reported in 2020), leading to the proposed classification of a type 2 SWS subtype in the OMIM database [16].

Clinical outcomes in SWS are poor during early life. Approximately 42% of patients die before the age of two, primarily from respiratory failure (71%) and autonomic dysfunction (67%) [1]. Hyperpyrexia in these patients can precipitate systemic inflammatory responses and multi-organ failure, similar to mechanisms observed in heat stroke. Therefore, recurrent unexplained fevers in neonates and infants – particularly when accompanied by joint anomalies, dysphagia, or respiratory distress – should raise suspicion of thermoregulatory genetic syndromes. Early genetic evaluation is crucial for timely diagnosis. Dysautonomia commonly presents as altered thermoregulation, diminished pain sensitivity, and abnormal sweating patterns, heightening the risk of dehydration and secondary complications. Although mortality considerably decreases after early childhood, survivors frequently contend with progressive orthopaedic complications necessitating long-term follow-up and surgical interventions [4,11].

Given the involvement of multiple organ systems in SWS, there is currently no specific treatment available for clinical management. However, it is crucial to enhance collaboration and follow-up among multidisciplinary teams. Physicians should conduct comprehensive assessments of patients’ diverse symptoms and develop personalised treatment plans to improve quality of life, reduce complications and enhance prognosis. Although the mortality rate among patients with SWS remains high, survivors generally exhibit a lower mortality rate beyond the age of two with unaffected cognitive abilities [10]. In terms of prospects, research into SWS should primarily concentrate on exploring the potential of gene therapy and personalised medicine while emphasising improvements in quality of life and treatment efficacy for patients with the syndrome.

## 4 Conclusion

In summary, SWS is a rare skeletal dysplasia characterised by dysautonomia and primarily caused by impaired LIFR signalling. Although LIFR variants are the principal known genetic cause, variants in related genes such as GP130 and CNTFR have been identified in syndromes with overlapping clinical features, underscoring the complexity of the molecular pathways involved. The syndrome is associated with a poor prognosis and currently lacks effective treatment options. Further research is essential to enhance our understanding of its underlying mechanisms. Molecular analysis plays a crucial role in distinguishing SWS from other disorders with similar clinical presentations but distinct genetic backgrounds, thereby facilitating the development of targeted therapies focused on specific signalling pathways.
